# Development of Optimized Ultrasound-Assisted Extraction Methods for the Recovery of Total Phenolic Compounds and Anthocyanins from Onion Bulbs

**DOI:** 10.3390/antiox10111755

**Published:** 2021-11-03

**Authors:** Ana V. González-de-Peredo, Mercedes Vázquez-Espinosa, Estrella Espada-Bellido, Marta Ferreiro-González, Ceferino Carrera, Gerardo F. Barbero, Miguel Palma

**Affiliations:** Department of Analytical Chemistry, Faculty of Sciences, Agrifood Campus of International Excellence (ceiA3), IVAGRO, University of Cadiz, Puerto Real, 11510 Cadiz, Spain; ana.velascogope@uca.es (A.V.G.-d.-P.); mercedes.vazquez@uca.es (M.V.-E.); estrella.espada@uca.es (E.E.-B.); marta.ferreiro@uca.es (M.F.-G.); ceferino.carrera@uca.es (C.C.); miguel.palma@uca.es (M.P.)

**Keywords:** *Allium cepa* L., anthocyanins, Box–Behnken, onion, phenolic compounds, UHPLC, ultrasound-assisted extraction

## Abstract

*Allium cepa* L. is one of the most abundant vegetable crops worldwide. In addition to its versatile culinary uses, onion also exhibits quite interesting medicinal uses. Bulbs have a high content of bioactive compounds that are beneficial for human health. This study intends to develop and optimize two appropriate ultrasound-assisted methods for the extraction of the phenolic compounds and anthocyanins present in red onion. A response surface methodology was employed and, specifically, a Box–Behnken design, for the optimization of the methods. The optimal conditions for the extraction of the phenolic compounds were the follows: 53% MeOH as solvent, pH 2.6, 60 °C temperature, 30.1% amplitude, 0.43 s cycle, and 0.2:11 g sample/mL solvent ratio. On the other hand, the optimal conditions for the anthocyanins were as follows: 57% MeOH as solvent, pH 2, 60 °C temperature, 90% amplitude, 0.64 s cycle, and 0.2:15 g sample/mL solvent ratio. Both methods presented high repeatability and intermediate precision, as well as short extraction times with good recovery yields. These results illustrate that the use of ultrasound-assisted extraction, when properly optimized, is suitable for the extraction and quantification of the compounds of interest to determine and improve the quality of the raw material and its subproducts for consumers.

## 1. Introduction

The genus *Allium* spans to more than 750 species that can be found all over the Northern Hemisphere [1]. Among these species, *Allium cepa* L. (common onion) is one of the most ancient crops cultivated worldwide and among the most popular ones [2]. Its great popularity is largely thanks to its versatile culinary uses as a raw food or in different cooked forms: baked, boiled, braised, grilled, fried, and so on [3]. In addition to its extended use as a flavored vegetable or spicy ingredient, onions are also well known for their employment in different forms of traditional medicine [4,5]. Numerous epidemiological studies have confirmed that the regular consumption of onions decreases the occurrence of various forms of cancer or cardiovascular and neurodegenerative diseases [2,6,7,8]. Onions are rich in antioxidant compounds, such that their consumption represents an interesting provision of antioxidants that contribute to prevent certain diseases associated with oxidative stress. From the article previously published by our research group [9], onion bulbs present a high content of flavonols, which are phenolic compounds with a high antioxidant capacity, as well as other health-promoting properties. However, flavonols are not the only compounds responsible for the antioxidant capacity of this bulb. Thus, in addition to flavonols, onions, particularly its red varieties, are a rich source of anthocyanins. Anthocyanins are natural pigments from the phenolic compounds family. Their interest comes from their capacity to directly scavenge reactive oxygen species (ROS) [10]. In fact, anthocyanin-rich vegetables have been demonstrated to have a health-promoting effect against several disorders or processes such as cancer, neurological diseases, inflammation, diabetes, or bacterial infections [11,12]. Furthermore, although vegetables have generally a lower anthocyanin content than most fruits, root and tuber types of vegetables (such as potatoes, carrots, and onions) present some advantages, such as their lower cost and longer storage periods, which favors a greater consumption [11]. Concerning onions, they have been frequently reported to contain cyanidin derivatives, together with some minor amounts of peonidin derivatives [13,14,15]. In fact, red onions varieties are the only ones reported to have a significant anthocyanin content, which, together with a greater total phenolic compounds content, implies that these varieties have higher antioxidant capacity [13]. The considerable intake of phenolic compounds and particularly of anthocyanins would make red onions particularly healthy food [16].

For these reasons, a rapid and efficient extraction and analysis method needs to be developed to obtain quality extracts of these bioactive compounds from onions. However, analyzing anthocyanins is a difficult task because of their liability to alkaline pH, light, or temperature [17]. Therefore, to prevent their degradation, the extraction procedure should be as short as possible. Traditionally, anthocyanins have been extracted from onions by means of organic solvents, such as methanol, using long processes at low temperature [18]. Table 1 describes some of the extraction methods that have been used by other authors to extract anthocyanins from onions. Most of the methods involve long periods or shorter periods of stirring, but repeated several cycles to obtain relevant yields. This implies a large consumption of solvents and, therefore, an increment in costs. In this study, ultrasound-assisted extraction (UAE) is presented to improve the methods for the extraction of both anthocyanins and phenolic compounds from red onion. UAE, supported by the phenomenon of cavitation, achieves a greater dispersion of the solid phase into the liquid and enhances contact interface [19]. This means that greater yields can be obtained in a shorter time, thus reducing solvent consumption and costs, which makes UAE a more environmentally friendly technique [20]. Nevertheless, although the use of UAE represents on its own an improvement when compared with traditional techniques, the optimization of several key parameters associated with performance is also a crucial aspect regarding the efficiency. Currently, temperature, time, solvent type, and concentration are some of the variables that greatly affect UAE efficiency [21,22,23].

Response surface methodology (RSM) is one of the experimental designs most often used for the optimization of the variables involved in extraction processes. One of the advantages of RSM lies in the fact that it allows the evaluation of the actual effect that multiple factors as well as their interactions have on one or more response variables. For this study, a Box–Behnken (BBD) design was chosen because it allows generating higher-order response surfaces using fewer runs than a regular factorial technique [24]. In addition to studying individually the effect on the UAE process from each one of the response variables, a multi-response optimization (MRO) approach with desirability functions was also applied. The possibility of performing a simultaneous analysis of both phenolic compounds and anthocyanins in red onions is also of great interest because of the cost and time saving that this represents.

Having efficient extraction methods would be quite useful, as it should facilitate the extraction and quantification of the compounds of interest for the different industries and would allow better quality raw material and its byproducts to be supplied to consumers. Thus, the concrete aim of the present study is the development of optimized individual and combined UAE methods for the extraction of phenolic compounds and anthocyanins from red onions. The general objective is to highlight how the use of UAE, an advanced extraction technique, duly optimized by RSM and MRO, leads to valuable improvements in relation to yield and extraction times.

**Table 1:** Extraction methods used by other authors to extract anthocyanins from onions.

Publication Year	Extraction Method	Anthocyanins Analyzed ^1^	Onion Variety	Total Anthocyanins Measured (mg g^−1^)	Reference
2020	Homogenization (1 min), sonication (30 min), and centrifugation. The supernatant residue was re-extracted twice	1, 2, 3, 4, 5, 6, 7	Honeysuckle red onions and sweet Italian red onions	Honeysuckle 0.103 ± 2.206 Sweet Italian 0.086 ± 1.843	[25]
2019	Centrifugation at 3214 g. The supernatant residue was re-extracted until the samples turned colourless	1, 2, 4, 5	Red onion	0.056	[26]
2018	Sonication at 60 °C for 1 h	Total anthocyanins measured by colourimetric methods	Red onions from eight different cultivars	0.02 ± 0.01–0.12 ± 0.01	[27]
2018	Three different methods: maceration (24 h), percolation (8 h), reflux and Soxhlet method (2 h). The extractions were repeated three times	1, 4, 5, 8	Bima Brebes and Maja Cipanas	Maceration: 1.463 ± 0.013 and 1.181 ± 0.008 Percolation: 0.328 ± 0.010 and 0.597 ± 0.015 Reflux: 1.415 ± 0.08 and 1.449 ± 0.013 Soxhlet: 0.218 ± 0.021 and 0.342 ± 0.022	[28]
2017	Sonication in an ultrasonic bath at 4 °C for 24 h	1, 4, 11, 12, 13	Dark-red onion cultivar ‘Xiu Qiu’ and white onion cultivar ‘Ring Master’	Xiu Qui: 0.3587 ± 0.0054 Ring Master: 0.0142 ± 0.0087	[29]
2013	Extraction at 4 °C, overnight or for 2 h	Total anthocyanins measured by colourimetric methods	Red onion	0.9966	[30]
2012	The extraction was carried out on a rotary shaker overnight (15 h; 400 rpm) at room temperature	1, 4, 9, 14	Red onion Pier-C and Red onion Pearl	Red onion Pier-C: 0.0777 ± 0.0038 Red onion Pearl: 0.1895 ± 0.0363	[11]
2011	Centrifugation at 1200 rpm (3 min) and agitation (15 min). Each homogenate was extracted three	1, 2, 4, 5, 6	Red onion “Vermelha da Povoa”	0.059	[31]
2011	Shaking (15 min) and centrifugation. Two additional extractions were performed for each sample	1, 2, 4, 5, 6	Red onion “Vermelha da Povoa”	0.003 ± 0.016	[32]
2010	Incubating (1 h) at room temperature with alternative shaking and subsequently centrifuged at 4000 rpm for 15 min at 28 °C. Two additional extractions were performed	1, 2, 4, 5, 6, 9, 10, 15	Red Onion Vermelha da Povoa, improved Vermelha da Povoa and Red Creole	Vermelha da Povoa: 0.057 ± 0.018 Improved Vermelha: 0.128 ± 0.046 Red Creole: 0.286 ± 0.08	[14]

^1.^ Compounds analyzed: 1. cyanidin 3-*O*-glucoside, 2. cyanidin 3-*O*-laminaribioside, 3. delphinidin 3,5-*O*-diglucoside, 4. cyanidin 3-*O*-(6″-malonoylglucoside), 5. cyanidin 3-*O*-(6″-malonoyl-laminaribioside), 6. Peonidin 3-*O*-malonoylglucoside, 7. cyanidin 3-*O*-(malonoyl)-(acetyl)-glucoside, 8. cyanidin 3-*O*-arabinoside, 9. cyanidin 3-*O*-(3″-malonilglucósido), 10. pedonidina 3-*O*-glucósido, 11. delphinidin 3-*O*-diglucoside, 12. delphinidin 3-*O*-glucoside, 13. delphinidin aglycon, 14. cyanidin 3-*O*-(malonyl)diglucoside, 15. cyanidin 3-*O*-dimalonylaminaribioside.

## 2. Materials and Methods

### 2.1. Biological Material

A stock of red onions was purchased in 2019 from a local market in the province of Cadiz (Spain) to be used as the biological material for this study. More specifically, the bulbs of those red onions were to be used for the analysis. For that purpose, after peeling the hard outer skin from each onion bulb, their pulp was chopped into small pieces using a knife. The chopped onions were lyophilized by means of an LYOALFA freeze dryer (Azbil Telstar Technologies, Terrasa, Barcelona, Spain) and crushed using a knife mill GRINDOMIX GM 200 (Retsch GmbH, Haan, Germany) to finally obtain <300 μm particles. This homogeneous material was stored in a freezer at −20 °C before analysis.

### 2.2. Chemical Reagents

The extraction solvents employed in this study were mixtures of methanol (Fischer Chemical, Loughborough, UK) of HPLC purity and Milli-Q water, obtained from a Milli-Q water purification system (Millipore, Bedford, MA, USA), with different pH values. A sodium hydroxide solution (NaOH, 1 M) and a hydrochloric solution (HCl, 1 M), both from Panreac, Barcelona (Spain), were used to adjust pH.

A variety of chemicals were used to determine bioactive compounds’ content. According to the Folin–Ciocalteau spectrophotometric method, anhydrous sodium carbonate (Panreac Química, Castellar del Valles, Barcelona, Spain) and Folin–Ciocalteu (Merck KGaA, EMD Millipore Corporation, Darmstadt, Germany) were employed to measure total phenolic compounds’ content. Regarding UHPLC analyses, methanol (Fischer Scientific, Loughborough, UK), Milli-Q water, and formic acid (Scharlau, Barcelona, Spain) were used to determine anthocyanins’ content. According to the DPPH assay, DPPH (2,2-diphenyl-1-picrylhydrazyl) radical scavenging (Sigma-Aldrich, San Luis, MO, USA) was used. The standard used for the phenolic compounds was gallic acid, the standard for anthocyanins was cyanidin chloride, and the standard for antioxidant activity was 6-hydroxy-2,5,7,8-tetramethylchroman-2-carboxylic acid (Trolox), all supplied by Sigma-Aldrich Chemical Co. (St. Louis, MO, USA). In all the analyses the extracts were previously filtered through a nylon filter (Membrane Solutions, Dallas, TX, USA) of 0.45 µm for Folin–Ciocalteau and DPPH assay, and of 0.2 µm for UHPLC analysis.

### 2.3. Extraction of Bioactive Compounds

As mentioned above, the extraction of bioactive compounds (anthocyanins and total phenolic compounds) from red onions was carried out in this study by ultrasound-assisted extraction. Specifically, a Sonopuls HD 2070.2 processor, 20 Hz (BANDELIN electronic GmbH & Co KG, Heinrichstrabe, Berlin, Germany), which allows controlling the cycle, the amplitude, and the working time, was employed. An adjustable double vessel thermostatic bath with temperature control was also used (Frigiterm-10, Selecta, Barcelona, Spain). With respect to the UAE probe, a versus 70 T (BANDELIN electronic GmbH & Co KG, Heinrichstrabe, Berlin, Germany) with the following characteristics: approximately 130 mm in length, 13 mm in diameter, 13 µm in amplitude, and 20–900 mL in volume, was used.

Regarding the experimental protocol applied, about 0.2 g of the lyophilized and homogenized sample was weighed in a Falcon tube and the corresponding solvent volume (at the specific pH and methanol/water ratio) was added. The Falcon tube was placed into the double vessel to maintain the sample at the desired temperature, and the ultrasound probe was submerged into it. The solvent type and volume, the temperature, the cycle and the amplitude were set according to each experiment requirement. The range of UAE conditions for the extractions was as follows: % methanol in water 50–100%, temperature 10–60 °C, amplitude 30–90% of the equipment maximum power (70 W), cycle 0.4–1 s, pH 2–7, and sample/solvent ratio of 0.2:10–0.2:20 g sample/mL solvent. The initial extraction time was the only parameter that was set to a constant value of 10 min for all of the experiments, which was followed by a sample cooling time. The extracts obtained were then centrifuged at 1702× *g* for 5 min. The supernatant was collected, and the precipitate was re-centrifuged under the same conditions after adding 5 mL of the same extraction solvent. The two supernatants that were obtained were mixed together and transferred to a volumetric flask (25 mL), which was filled up with the same solvent. The extracts were kept at −20 °C for subsequent analysis.

### 2.4. Analysis of Bioactive Compounds

#### 2.4.1. Analysis of Total Phenolic Compounds

The total phenolic compounds (TPCs) content in red onion was determined by means of a modified Folin–Ciocalteau (FC) method [33]. Folin–Ciocalteu is a colorimetric method based on the fact that phenolic compounds react with Folin–Ciocalteu reagent (a mixture of sodium tungstate and sodium molybdate) with basic pH, which gives rise to a blue color susceptible to be determined spectrophotometrically at 765 nm. Specifically, the FC assay was performed by transferring 0.25 mL of UAE onion extract, 1.25 mL of water, and 1.25 mL of the Folin–Ciocalteu reagent into a 25 mL volumetric flask. Then, 5 mL of aqueous sodium carbonate solution (20% p/v) was also added, and the solution was made up to the mark with water. After 30 min, the solution absorbance was measured at 765 nm. The absorbance was measured on a Cary 4000 UV/Vis (Agilent, Santa Clara, CA, USA). Gallic acid was used as the standard. Therefore, the results are expressed as milligrams of gallic acid equivalent per g of dry weight (mg GAE g^−1^ DW). The linear regression of the standard was constructed using six points (50–0.5 mg L^−1^) in triplicate. The regression equation (y = 0.0014x + 0.0022) and the determination coefficient (R^2^ = 0.9995) were calculated by means of Microsoft Office Excel 2013.

#### 2.4.2. Identification of the Anthocyanins

The anthocyanin content in red onion was determined by liquid chromatography. Firstly, the anthocyanins in the UAE extracts were identified by ultra-high-performance liquid chromatography (UHPLC) coupled to a quadrupole time-of-flight mass spectrometer (Q-ToF-MS) (Xevo G2 QToF, Waters Corp., Milford, MA, USA). The chromatographic separation was performed on a reverse-phase C18 analytical column (1.7 µm, 2.1 mm × 100 mm, made by Acquity UPLC BEH C18, Waters Corp., Milford, MA, USA). The gradient of the UHPLC-Q-ToF-MS method was as follows (time, % solvent B): 0.00 min, 15%; 3.30 min, 20%; 3.86 min, 30%; 5.05 min, 40%; 5.35 min, 55%; 5.64 min, 60%; 5.94 min, 95%; and 7.50 min, 95%. The flow rate was 0.4 mL min^−1^, the injection volume was 3.0 μL, and the mobile phase was a binary solvent system (2% formic as phase A acid and methanol as phase B). An electrospray operating in positive ionization mode was used to perform the analyses under the following conditions: desolvation gas flow = 700 L h^−1^, desolvation temperature = 500 °C, cone gas flow = 10 L h^−1^, source temperature = 150 °C, capillary voltage = 700 V, cone voltage = 30 V, and collision energy = 20 eV. The full-scan mode was used (*m*/*z* 100–1200). The following nine anthocyanins were individually identified based on their retention time and molecular weight: cyanidin 3-*O*-glucoside (3.517 min, *m*/*z* 449.1087), cyanidin 3-*O*-laminaribioside (4.132, *m*/*z* 611.1641), cyanidin 3-*O*-(3″-malonylglucoside) (4.875 min, *m*/*z* 535.1069), peonidin 3-*O*-glucoside (5.384 min, *m*/*z* 463.1251), delphinidin 3,5-*O*-diglucoside (5.721, *m*/*z* 649.1392), cyanidin 3-*O*-(6″-malonylglucoside) (5.850, *m*/*z* 535.1104), cyanidin 3-*O*-(6″-malonyl-laminaribioside) (6.052 min, *m*/*z* 697.1613), peonidin 3-*O*-(6″-malonylglucoside) (6.323 min, *m*/*z* 549.1255), and delphinidin 3-*O*-glucoside (6.536, *m*/*z* 487.0863). The data regarding the anthocyanins identified in the samples and their mass spectrum are included in the Supplementary Material.

#### 2.4.3. Analysis of the Anthocyanins

Once the anthocyanins were identified, an Elite UHPLC LaChrom Ultra System (Hitachi, Tokyo, Japan) was used to separate and quantify them. The UHPLC system is equipped with an L-2420U UV/Vis detector, an L-2200U autosampler, an L-2300 column oven, and two L-2160 U pumps. The chromatographic separation was performed on a reverse-phase C18 analytical column (2.6 µm, 2.1 mm × 100 mm, made by Phenomenex, Torrance, CA, USA). The gradient and characteristics of the UHPLC method employed in this work were previously published by our research group [34]. Cyanidin chloride was used as the standard to quantify the seven anthocyanins identified in onions. The linear regression for the standard was constructed using six points in triplicate (0.06–35 mg L^−1^). The regression equation (y = 260,596.88x − 4292.66) and the determination coefficient (R^2^ = 0.9999) were also calculated using Microsoft Office Excel 2013. Using the same software, the limit of detection (LOD) (0.113 mg L^–1^) and the limit of quantification (LOQ) (0.402 mg L^–1^) were also calculated. Repeatability was also studied using nine replicates on the same day. Specifically, it was evaluated in terms of retention time and area of each of the anthocyanin peaks. The results, expressed as the coefficient of variance (CV), were all less than 10%, which is the acceptable CV limit according to the AOAC manual for peer-verified methods. Specifically, the repeatability takes values within the range of 0.05–0.14% for retention time and 0.59–7.26% for areas.

**Figure 1:**
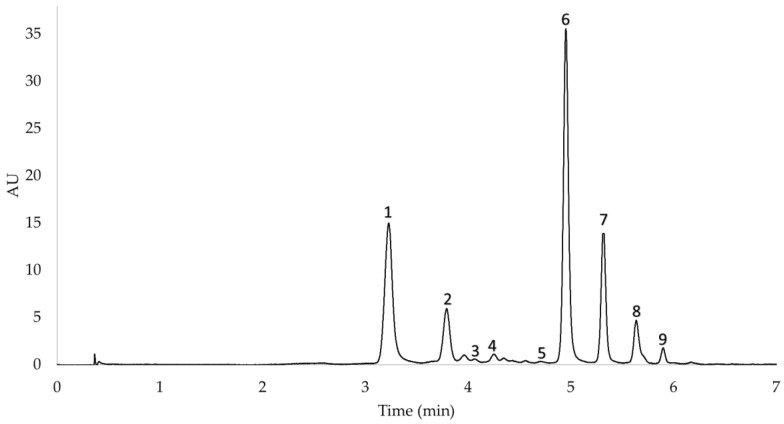
Anthocyanins identified in red onion. Chromatograms peaks corresponding to the nine anthocyanins identified in the UAE extracts from red onion samples. Peak 1. cyanidin 3-*O*-glucoside, peak 2. cyanidin 3-*O*-laminaribioside, peak 3. cyanidin 3-*O*-(3″-malonylglucoside), peak 4. peonidin 3-*O*-glucoside, peak 5. delphinidin 3,5-*O*-diglucoside, peak 6. cyanidin 3-*O*-(6″-malonylglucoside), peak 7. cyanidin 3-*O*-(6″-malonyl-laminaribioside), peak 8. peonidin 3-*O*-(6″-malonylglucoside), peak 9. delphinidin 3-*O*-glucoside.

Once the regression equation of cyanidin chloride was obtained, a calibration curve was plotted for each anthocyanin that was identified in the onion samples. For this purpose, it was assumed that the nine anthocyanins have similar absorbance, and the molecular weight of each anthocyanin was taken into account. The results were expressed as milligrams of each anthocyanin per g of dry weight (mg g^−1^ DW). The final UHPLC chromatogram can be seen in Figure 1.

#### 2.4.4. Determining Antioxidant Activity

A number of different techniques to assess the antioxidant activity of food and plants have been described in the literature. However, preferential attention has been given to the technique that uses 2,2-diphenyl-1-picrylhydrazyl free radical, better known by its acronym DPPH. For this assay, the procedure designed by Brand-Williams et al. [35] and modified by Miliauskas et al. [36] was employed. First, a 6 × 10^−5^ M DPPH solution was prepared in methanol. Then, for each 100 μL of onion extract, 2 mL of the DPPH solution was added to the mixture. The mixture was incubated for 40 min in the absence of light and at room temperature. Then, the absorbance was measured at 515 nm. The results were expressed as mg of Trolox equivalents (TE) per g of dry weight sample. For this purpose, a Trolox calibration curve (y = 88.94x + 0.75; R^2^ = 0.9959) was plotted using six points (0–1.4 mM) in triplicate.

### 2.5. Applying Box–Behnken Design to Optimize the UAE Methods

For the development and optimization of the UAE methods, the spherical response surface Box–Behnken design (BBD) was applied. The Box–Behnken design is characterized by the factor levels being placed at the midpoints of the edges and at the centre of the space [37]. This implies that fewer data points are required when compared with other designs. For example, unlike the central composite design, BBD does not have corner points. This also means that the factors are in no case either all high or all low at the same time, i.e., no extreme combinations take place [38]. Thus, in a BBD, every factor has three levels: a lower level (−1), an intermediate level (0), and an upper level (1) [29].

In this work, six independent factors were considered within the following ranges: composition of the solvent (% methanol in water) (*X*_1_: 50, 75, 100 °C), pH of the solvent (*X*_2_: 2, 4.5, 7), extraction temperature (*X*_3_: 10, 35, 60 °C), ultrasound amplitude (*X*_4_: 30, 60, 90%), ultrasound cycle (*X*_5_; 0.4, 0.7, 1 s), and sample mass/solvent volume ratio (*X*_6_: 0.2:10, 0.2:15, and 0.2:20 g sample/mL solvent). All of these ranges were selected for the study based on the group’s previous experience [9,21,23,39]. Thus, the values within these ranges allow performance optimization while no degradation takes place. Temperature was the only factor that was studied separately because phenolic compounds, and especially anthocyanins, may present instability when subjected to high temperature levels [40,41].

**Figure 2:**
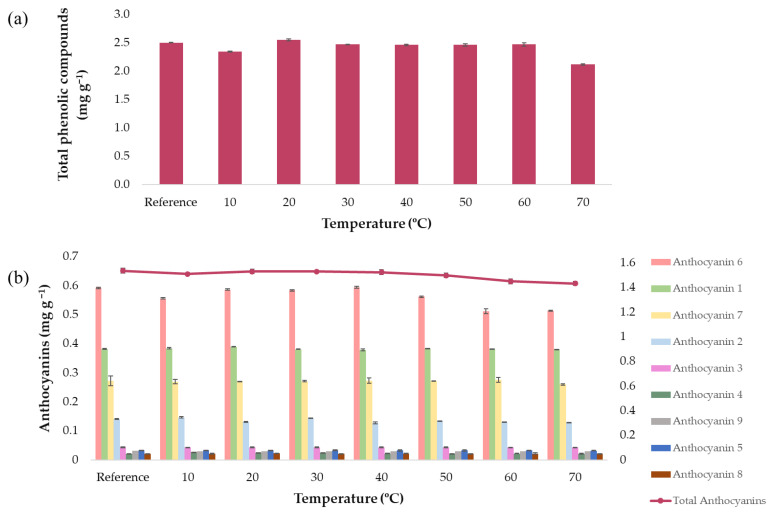
Extract content stability at different temperatures (*n* = 3): (**a**) total phenolic compounds content and (**b**) individual and total anthocyanins, in red onion extracts.

In order to determine the temperature for the extractions, several runs were conducted at different temperatures according to the protocol previously explained in Section 2.2. First of all, a control extract was obtained by carrying out an extraction under intermediate conditions (50:50 MeOH/H_2_O extraction solvent, 60% ultrasound amplitude, 0.5 s cycle, 0.2:15 g sample/mL solvent, and 20 min extraction time) where no external heat source was applied. Then, 15 mL of the control extract was acquired and subjected to different temperature levels, while the rest of the variables remained constant at the aforementioned intermediate conditions. This procedure was repeated at each one of the temperature levels considered for the study, i.e., 10, 20, 30, 40, 50, 60, and 70 °C. The anthocyanins and phenolic compounds yields obtained under each temperature can be seen in Figure 2a,b.

It can be observed from Figure 2a how the total phenolic compounds’ content is lesser at a temperature of 70 °C, while at lower temperature levels, no significant differences in the concentration of bioactive compounds can be noticed. Regarding anthocyanins (Figure 2b), both individual anthocyanins and total anthocyanins were represented, in order to know if there are opposite tendencies depending on each anthocyanin studied. As for phenolic compounds, the total anthocyanins content was lesser at 70 °C and remained constant at the lowest temperature levels. This decrease in the content of total anthocyanins is mainly due to the following anthocyanins that showed a decrease in their content as the temperature increased: cyanidin 3-*O*-(6″-malonylglucoside), cyanidin 3-*O*-(6″-malonyl-laminaribioside), and cyanidin 3-*O*-glucoside. Other anthocyanins such as cyanidin 3-*O*-(3″-malonylglucoside) or peonidin 3-*O*-(6″-malonylglucoside) kept their content constant in all the temperatures evaluated. Based on these results, a temperature range from 10 to 60 °C would be used, because, only at temperatures over 60 °C, a decrease in the concentration of both compounds, probably owing to the aforementioned degradation, could be registered.

With regard to the response variables, two responses were considered for this study as follows: total phenolic compounds (TPC) in red onion bulbs, determined by the Folin–Ciocalteau method (*Y_TPC_*, mg g^−1^); and total anthocyanins (TA) in red onion bulbs (*Y_TA_*, mg g^−1^), calculated as the sum of concentrations corresponding to each one of the nine individual anthocyanins quantified by UHPLC. Finally, and according to the specific BBD equation, a design comprising 54 extraction runs, including six repetitions at their centre point to determine the error, was obtained. All the experiments were carried out at random. These experiments and the resulting data can be seen in Table 2.

The advantage of RSM is that it can reduce the prediction error and improve the estimate by means of a polynomial equation [42]. The results from this second-order polynomial equation (Equation (1)) match as closely as possible the actual experimental responses according to the corresponding conditions.

(1) $$Y=\beta_{0}+\overset{k}{\underset{i=1}{\sum }}\beta_{i}X_{i}+\beta_{ii\;}X_{i}^{2}+\underset{i}{\sum }\overset{k}{\underset{i=1}{\sum }}\beta_{ij}X_{i}X_{j}+r$$

In this equation, *Y* represents the responses (*Y_TPC_* and *Y_TA_*); *β_0_* is the model constant; *X* represents each one of the factors considered; *β_i_* is the coefficient of each main effect; *β_ii_* is the coefficient of the quadratic factors that represent the curvature of the surface; *β_ij_* is the coefficient corresponding to the interactions between factor *i* and factor *j*; and *r* is the residual value (random error). The statistical significance of the polynomial model and the regression terms were evaluated by applying an analysis of variance (ANOVA) following a similar protocol to the one used by Jadhav S.B. et al. [43]. Specifically, the *F*-test and the ‘lack of fit’ test were evaluated using the software applications Statgraphics Centurion version XVI (Warrenton, VA, USA) and Design Expert (Version 13, Stat-Ease Inc., Minneapolis, MN, USA).

### 2.6. Multi-Response Optimization by Desirability Functions

This research work was focused on optimizing two response variables, the extraction of total phenolic compounds and the extraction of anthocyanins. As an alternative to the individual optimization of each response variable, multi-response optimization was proposed. The desirability function is one of the methods that is most frequently used to perform multi-response surface optimizations. To apply the desirability function approach, each estimated response is transformed into a scale-free value within the range 0 ≤ *d_i_* ≤ 1. This is known as desirability (*d_i_*). The overall desirability function *D* (Equation (2)) is defined as the geometric average of the individual desirability functions of each response *d_i_* (*Y_i_*), where *m* is the number of responses. The optimal solutions are determined by maximizing *D*.

*D* = (*d*_1_ × *d*_2_ × … *d_m_*)^1/*m*^, (2)

The software application Statgraphics Centurion version XVI (Warrenton, VA, USA) was used to statistically analyze the results obtained by means of each separately and by the multi-response optimization design. Given that according to Shapiro–Wilk test, the resulting values follow a normal distribution (*p*-value < 0.05), and that according to Levene’s test, they present the same variance (*p*-value < 0.05). An ANOVA test was carried out to detect any statistically significant differences (5% level of significance) between the means obtained by each one of the two separate methods and the combined method.

## 3. Results and Discussion

### 3.1. Developing a UAE Method for Total Phenolic Compounds by Means of a Box–Behnken Design

After the experimental matrix was completed (Table 2), an ANOVA was applied to evaluate the effect of the factors and the possible interactions between them. The results from the ANOVA are shown in Table 3. Based on these results, it can be confirmed that the analysis explains 82.50% of the total variability. In addition, to demonstrate the validity of the polynomial model, the ANOVA indicates the coefficients of the different parameters in the quadratic polynomial equation and their significance (*p*-values). According to such significance, the factors and/or interactions with a more relevant influence on the response can be determined. Thus, only those factors and/or interactions with *p*-values lower than 0.05 were considered to have a relevant influence on the response at the established level of significance (95%).

Regarding total phenolic compounds (Table 3), the linear terms were not significant (*p*-value > 0.05). With respect to quadratic interaction, the amplitude–amplitude (*X*_3_*X*_2_, *p*-value < 0.0001) and temperature–temperature (*X*_2_*X*_2_, *p*-value 0.01) interactions showed a relevant effect on the response. Finally, the following factor interactions were also determined as significant with *p*-values lower than 0.05: percentage methanol–amplitude (*X*_1_*X*_3_, *p*-value 0.00), percentage methanol–cycle (*X*_1_*X*_4_, *p*-value 0.00), percentage methanol–temperature (*X*_1_*X*_2_, *p*-value 0.01), temperature–ratio (*X*_2_*X*_6_, *p*-value 0.02), and amplitude–cycle (*X*_3_*X*_4_, *p*-value 0.02). Based on these results, it can be concluded that, except for pH, the rest of the variables have a relevant influence on the extraction of phenolic compounds from red onions samples. This highlights the importance of carrying out an experimental design when intending to extract bioactive compounds from natural matrices.

With regard to the quadratic interaction, amplitude–amplitude showed a positive effect (*b*_3_^2^ = 0.88) on the response variable. Amplitude is an important variable regarding extraction, as the energy provided by the ultrasounds is necessary to release the target compounds from the matrix [40]. The interaction of temperature–temperature also showed a positive effect (*b*_2_^2^ = 0.35) on the response variable. The temperature is an important variable; for a successful extraction, the temperature must be sufficient to favor the solubility, diffusion, and transfer of the compounds of interest in the solvent, but not so high as to produce degradation [44].

**Figure 3:**
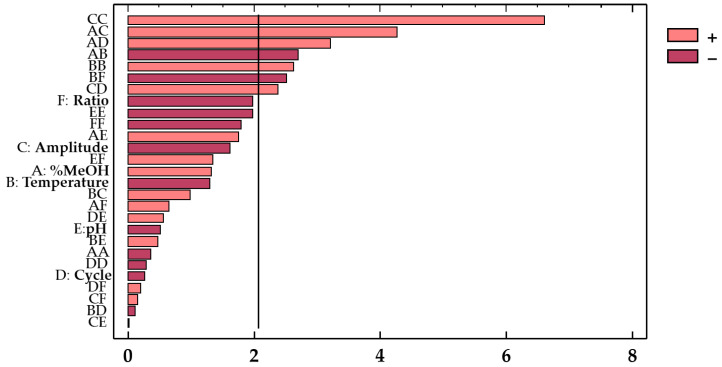
Standardized Pareto chart of the total phenolic compounds from red onion extracts.

For a better understanding, a Pareto chart (Figure 3) was included to illustrate the influence from each factor and combination of factors on the response. The effect from each factor or factor interaction is graphically represented by bars arranged in decreasing order of influence on the response.

Finally, based on the coefficients of the factors and interactive effects (Table 3), a polynomial equation can be obtained to predict total phenolic compounds’ content (response variable, *Y_TPC_*) as a function of the independent variables (Equation (3)). The full equation could be reduced by considering just the significant factors and interactions (*p*-value < 0.05). A reduced equation is represented in (Equation (4)).

*Y_TPC_* (mg g^−1^) = 2.57 + 0.11·*X*_1_ − 0.11·*X*_2_
*−* 0.14·*X*_3_ − 0.023·*X*_4_ − 0.045·*X*_5_ − 0.17·*X*_6_ − 0.046·*X*_1_^2^ − 0.41·*X*_1_*X*_2_ + 0.65·*X*_1_*X*_3_ + 0.34·*X*_1_*X*_4_ + 0.26·*X*_1_*X*_5_ + 0.098·*X*_1_*X*_6_ + 0.35·*X*_2_^2^ + 0.15·*X*_2_*X*_3_ − 0.015·*X*_2_*X*_4_ + 0.050·*X*_2_*X*_5_ − 0.38·*X*_2_*X*_6_ + 0.88·*X*_3_^2^ + 0.36·*X*_3_*X*_4_ − 0.0031·*X*_3_*X*_5_ + 0.016·*X*_3_*X*_6_ − 0.037·*X*_4_^2^ + 0.086·*X*_4_*X*_5_ + 0.031·*X*_4_*X*_6_ − 0.26·*X*_5_^2^ + 0.20·*X*_5_*X*_6_ − 0.24·*X*_6_^2^, (3)

*Y_TPC_* (mg g^−1^) = 2.57− 0.41·*X*_1_*X*_2_ + 0.65·*X*_1_*X*_3_ + 0.34·*X*_1_*X*_4_ + 0.35·*X*_2_^2^ − 0.38·*X*_2_*X*_6_ + 0.88·*X*_3_^2^ + 0.36·*X*_3_*X*_4_. (4)

**Figure 4:**
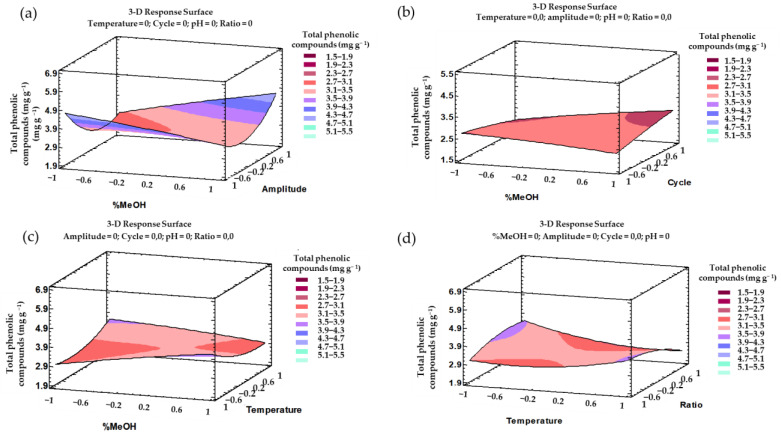
3D surface graphs of the Box–Behnken design according to the polynomial equations representing the effects from the different interactions on the total phenolic compounds extractions: (**a**) %MeOH and amplitude; (**b**) %MeOH and cycle; (**c**) %MeOH and temperature; and (**d**) temperature and ratio.

According to the fitted model based on the trends outlined above, a three-dimensional (3D) surface can be plotted. This 3D (Figure 4) graph facilitates the comprehension of the effect from the interactions of the most influential parameters, i.e., %MeOH–amplitude, %MeOH–cycle, %MeOH–temperature, or temperature–ratio on the total phenolic compounds’ recovery.

### 3.2. Developing a UAE Method for Total Anthocyanins Using a Box–Behnken Design

As for total phenolic compounds, an ANOVA was applied to the anthocyanin data matrix (Table 2), showing that the analysis explains 94.50% of the total variability. The rest of the results obtained are shown in Table 4.

Regarding total anthocyanins (Table 4), both the linear term percentage of methanol (*X*_1_, *p*-value < 0.0001) as well as the linear term pH (*X*_5_, *p*-value < 0.0001) exhibited a relevant effect on the response. Concerning the quadratic interactions, the percentage of methanol–percentage of methanol (*X*_1_*X*_2_, *p*-value < 0.0001), the pH–pH (*X*_5_*X*_2_, *p*-value 0.00), and the cycle–cycle (*X*_4_*X*_2_, *p*-value 0.03) interactions also showed a relevant effect on the response. Finally, the following factor interactions were also significant with *p*-values lower than 0.05: percentage methanol–temperature (*X*_1_*X*_2_, *p*-value 0.00) and percentage methanol–pH (*X*_1_*X*_4_, *p*-value 0.01).

**Figure 5:**
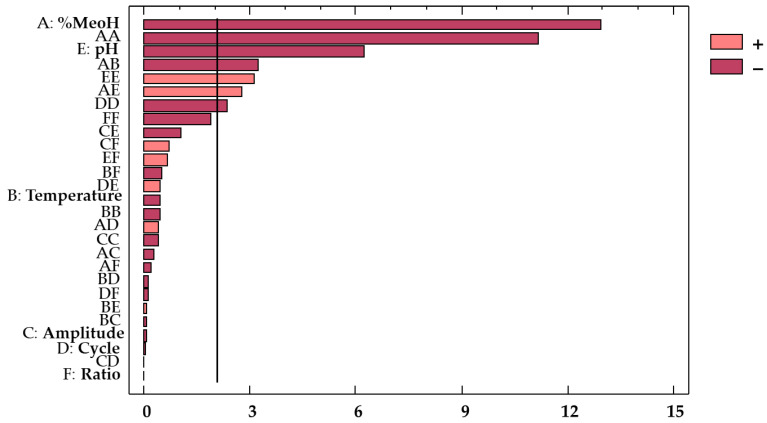
Standardized Pareto chart of total anthocyanins from red onion extracts.

Based on these results, it can be concluded that methanol and pH were the most significant variables regarding their influence on the anthocyanins’ extraction. In fact, both variables showed a negative effect (*b*_1_ = −0.47 and *b*_5_ = −0.23, respectively) on the response variable. The solvent composition plays an important role in the extraction of the bioactive compounds because a similar polarity between anthocyanins and solvent is required for a successful extraction. Owing to the negative effect of this factor (*b*_1_ = −0.47), when the solvent takes low polarity values within the range of the study, the variable shows the opposite effect, and a greater efficiency of the extraction is achieved. In this case, as the range goes from 50 to 100%, the results confirm that hydroalcoholic mixtures are more efficient than pure solvents (100% MeOH) for the extraction of amphiphilic or other moderately polar molecules, such as polyphenols. This had already been reported by other authors [12]. Likewise, pH also plays an important role in the bioactive compounds’ extraction process, and particularly in the extraction of anthocyanins, as they are more stable when pH remains within the range of 1 to 3 [41]. The acids in the solvents contribute to breaking down the cell membranes, thus improving the release and solubilization of the different compounds, such as anthocyanins [41]. This is clearly visualized in the Pareto chart (Figure 5).

Finally, the full polynomial equation (Equation (5)) and the reduced polynomial equation (Equation (6)) to predict the content of total anthocyanins (response variable, *Y_TA_*) as a function of the independent variables are included below.

*Y_TA_* (mg g^−1^) = 1.72 − 0.47·*X*_1_ − 0.016·*X*_2_ − 0.0024·*X*_3_ − 0.00075·*X*_4_ − 0.23·*X*_5_ + 0.00027·*X*_6_ − 0.63·*X*_1_^2^ − 0.20·*X*_1_*X*_2_ − 0.019·*X*_1_*X*_3_ + 0.019 ·*X*_1_*X*_4_ + 0.17·*X*_1_*X*_5_ − 0.014·*X*_1_*X*_6_ − 0.025·*X*_2_^2^ −0.0043·*X*_2_*X*_3_ − 0.0076·*X*_2_*X*_4_ + 0.0040·*X*_2_*X*_5_ − 0.032·*X*_2_*X*_6_ − 0.023·*X*_3_^2^ + 0.00056·*X*_3_*X*_4_ − 0.0660788·*X*_3_*X*_5_ + 0.032·*X*_3_*X*_6_ − 0.13·*X*_4_^2^ + 0.029·*X*_4_*X*_5_ + 0.0073·*X*_4_*X*_6_ − 0.17·*X*_5_^2^ + 0.043·*X*_5_*X*_6_ − 0.10·*X*_6_^2^, (5)

*Y_TA_* (mg g^−1^) = 1.72 − 0.47·*X*_1_− 0.23·*X*_5_ − 0.62·*X*_1_^2^ − 0.20·*X*_1_*X*_2_ + 0.17·*X*_1_*X*_5_ − 0.13·*X*_4_^2^ − 0.17·*X*_5_^2^, (6)

**Figure 6:**
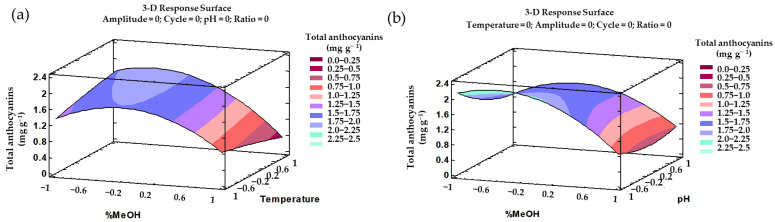
3D surface graphs of the Box–Behnken design according to the polynomial equations representing the effects from the different interactions on the total anthocyanin extractions: (**a**) %MeOH and temperature; (**b**) %MeOH and pH.

All the trends outlined above can be graphically represented in their corresponding 3D surface graphs (Figure 6). These 3D representations illustrate the effect from the most influential interactions, i.e., %MeOH–temperature and %MeOH–pH, regarding total anthocyanins’ recovery.

### 3.3. Optimal Conditions, Extraction Time, and Precision of the Two Developed Methods

Based on the Box–Behnken design, the optimum values that maximize the response variables, both the yields of total phenolic compounds and the yields of total anthocyanins, can be construed. Such optimal conditions that maximize the two responses separately and simultaneously are presented in Table 5.

According to the results obtained for both variables, 60 °C was selected as the optimal temperature. Higher temperatures were not considered as relevant degradation was observed in the stability study [40]. Regarding the solvent, an approximate concentration of 1:1 MeOH/H_2_O with acidic pH was selected as optimal for both variables. Each one of the components in a solvent mixture may play a specific role in the extraction. Thus, methanol increases the solubility of the bioactive compounds, while water contributes to the desorption of the solute from the sample. Therefore, a mixed solvent including both of these compounds should favor the extraction of phenolic compounds and anthocyanins [45]. Regarding pH, the extraction of the bioactive compounds is also promoted by acid solvents, as acid substances can break down cell membranes, which enhances the release and solubilization of the phenolic compounds and anthocyanins [46,47]. Amplitude was the factor that presented the greatest difference between both variables. The optimal amplitude for the extraction of phenolic compounds (30.1%) was close to the lower limit of the studied range, while for the extraction of anthocyanins, the optimal amplitude was near the upper limit (89.9%). This and other minor differences between each variable’s optimal values mean that not all the phenolic compounds extracted would be anthocyanins. In fact, other phenolic compounds present in onion matrices, such as quercetin derivatives, which is a flavonoid, can be found at high concentrations in onion bulbs. This has already been reported by our research group [9].

**Figure 7:**
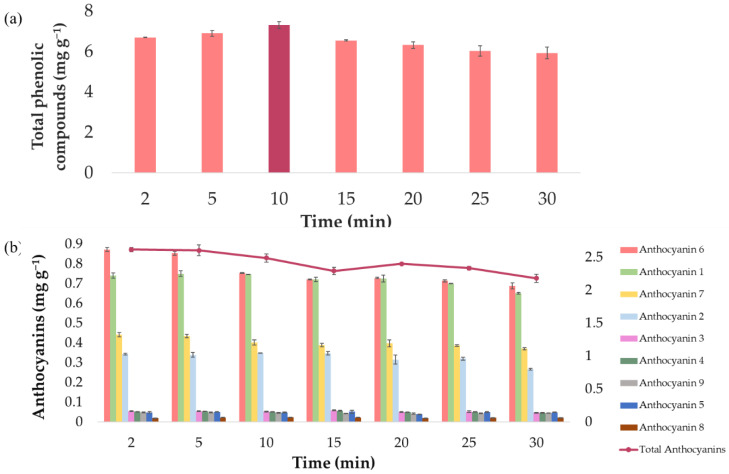
Average extraction yields (*n* = 3) resulting from different extraction times: (**a**) total phenolic compounds and (**b**) individual and total anthocyanins.

In addition to establishing the optimal values for the most relevant parameters, the optimal time for the extraction of the phenolic compounds and anthocyanins in onion bulbs was also determined. For this purpose, several extractions were carried out at different times, keeping the rest of the extraction parameters under the optimal conditions previously determined (Table 5). The times studied were as follows: 2, 5, 10, 15, 20, and 25 min. Each time was studied three times, and the average results (*n* = 3) for phenolic compounds and anthocyanins are displayed in Figure 7.

It can be concluded, from Figure 7a, that 10 min provides the best extraction of total phenolic compounds. In fact, an extraction time of 10 min allows 7.30 ± 0.015 mg g^−1^ phenolic compounds to be extracted, while shorter times (2 or 5 min) achieve lower yields, probably because the extraction process cannot be completed in such a short time. Similarly, longer times (>10 min) also yield less phenolic compounds, but in this case, it might be due to the degradation suffered by the phenolic compounds when they are subjected to ultrasounds for so long. With respect to total anthocyanins (Figure 7b), only 2 min is required to obtain the best yields (2.62 ± 0.034 mg g^−1^). In fact, after 5 min, the amount of anthocyanins extracted is practically the same. Therefore, 2 min was selected as the optimal extraction time, which represents a greater economic and time saving. Any time longer than 10 min resulted in lower extractions, probably owing to the already mentioned degradation of the phenolic compounds. Furthermore, individual anthocyanins were also represented, in order to know if there are opposite tendencies depending on each anthocyanin studied. All studied anthocyanins suffered a considerable decrease in their content when extraction times were greater than 10 min. Only the anthocyanins delphinidin 3,5-*O*-diglucoside (0.047 ± 0.004 mg g^−1^) and delphinidin 3-*O*-glucoside (0.044 ± 0.002 mg g^−1^) were unchanged with increasing time, showing no differences in their content as time increased.

Finally, the precision of the developed methods was evaluated in terms of repeatability and intermediate precision. For the assessment of their intermediate precision, 10 experiments were conducted on 3 consecutive days (a total of 30 experiments). Then, their intermediate precision was determined according to the coefficient of variation (CV) of the 30 experiments. Repeatability was determined by calculating the coefficients of variation of each of the 10 experiments completed on a single day. This method to determine the precision of a particular process has been typically employed in other works on similar natural matrices [18,42]. The percentages of repeatability (3.01% for TPC and 2.86% for TA) and intermediate precision (4.12% for TPC and 3.56% for TA) obtained were lower than 5%. Therefore, the UAE methods for the extraction of total phenolic compounds and total anthocyanins can both be considered to have good repeatability and intermediate precision, as 5% is the generally accepted variation limit [48].

### 3.4. Multi-Response Optimization and Application to Different Onion Varieties

In addition to individually optimizing each response variable, a multi-response optimization was carried out. The multi-response optimization was evaluated to determine the optimum balanced conditions to successfully obtain extracts with a high amount of both anthocyanins and phenolic compounds. The optimal conditions that simultaneously maximize both responses are presented in Table 5. The yields obtained when applying the optimal values from the multi-response study using an intermediate time of 10 min were 7.23 ± 0.034 mg g^−1^ for total phenolic compounds and 2.280 ± 0.081 mg g^−1^ for total anthocyanins. These results were slightly lower than those obtained when the specific optimal values for each method were applied also using an intermediate time of 10 min (7.30 ± 0.015 mg g^−1^ for total phenolic compounds and 2.49 ± 0.053 mg g^−1^ for total anthocyanins). Specifically, and assuming that the values follow a normal distribution and that there is no difference between the variances, it can be stated that there is a statistically significant difference between the means of the two variables with a significance level of 5%, as the *p*-value of the *F*-test is less than 0.05.

Despite this statistical difference, the yields obtained are not exceedingly different. Therefore, this could represent a practicable set of conditions for those cases where time and cost savings (using a single solvent, for example) are a priority. This method could probably be applied by quality control analytical laboratories, where time and costs must be minimized [21]. Consequently, this combined method was applied to a number of onion varieties in order to verify the efficacy of the multi-response method when applied to onions of diverse chemical composition.

For this purpose, a total of 24 types of onions were purchased from different supermarkets and greengrocers. The onions were of different colors, varieties, or origin. All of them were subjected to the same pretreatment in order to obtain a fine powder, and then the samples of such powder were extracted in triplicate using the multi-response optimized method. The extracts resulting from the different varieties were analyzed to determine their phenolic compounds and anthocyanins contents as well as their antioxidant activity. Table 6 includes the results obtained from these extractions as the mean of the three replicates ± the standard deviation.

As expected, the only extracts that contain anthocyanins are those from red/purple onion varieties, given that these compounds are largely responsible for their reddish coloration [49]. Although yellow and white onions do not contain anthocyanins in their matrices, they do contain total phenolic compounds with antioxidant activity. As previously published by our research group [9], these onion varieties contain in their matrices other phenolic compounds such as quercetin derivatives, which largely confer their antioxidant properties. Even so, it is logical that, in general, the content of total phenolic compounds and the antioxidant activity is higher in the red varieties (6.60 ± 1.45 mg g^−1^ and 6.28 ± 0.80 mg g^−1^, respectively) than in the yellow and white ones (4.51 ± 1.24 mg g^−1^ and 4.68 ± 1.22 mg g^−1^, respectively), as these onions present both anthocyanins as quercetin derivatives. The fact that practically only the red onions have been reported to have a significant anthocyanin content may open new fields of applications for these varieties (perfume, cosmetics, food industries, medicine, and so on).

Finally, it should be highlighted how the use of advanced extraction techniques, like the UAE method when properly optimized, leads to valuable improvements. For this purpose, the results obtained from this study against those reported by other authors (see the bibliographic review in Table 1), who employed traditional extraction techniques, were compared. Even the most recent publications [25,26,27] are related to traditional techniques such as homogenization, centrifugation, or sonication. Not only do these techniques require longer extraction times and a greater use of solvents, but also they give rise to lower yields than those actually achieved in this work. Specifically, R. Metrani et al. with 0.103 ± 2.206 mg g^−1^ anthocyanins yields, A. D. Front et al. with 0.056 mg g^−1^ anthocyanins yields, and M. J. Park et al. with 0.02  ±  0.01–0.12  ±  0.01 mg g^−1^ anthocyanins yields from similar onion matrices did not reach the amounts of anthocyanins obtained by applying the method developed in this study. It can, therefore, be concluded that UAE, when properly optimized by means of an experimental design and coupled to a chromatographic analysis technique, represents a valuable tool for the extraction of bioactive compounds from onions. To the best of our knowledge, this UAE method had not been developed until present. Suitable analytical techniques that allow to identify and quantify the compounds of interest that are present in the final product and, thereby, its quality are extremely interesting.

## 4. Conclusions

Two ultrasound-assisted extraction methods were developed and optimized to extract total phenolic compounds and anthocyanins from red onion samples. A Box–Behnken design was used to optimize the relevant process parameters and the following values were established for the extraction of phenolic compounds: extraction solvent 53% MeOH, pH 2.6, 60 °C temperature, 30.1% amplitude, 0.43 s cycle, and 0.2:11 g onion sample/mL solvent ratio. The optimal values for the extraction of anthocyanins were established as follows: extraction solvent 57% MeOH, pH 2, 60 °C, amplitude of 90%, cycle of 0.64 s, and ratio of 0.2:15 g sample/mL solvent. It was found that most of the studied variables influence the extraction of phenolic compounds and anthocyanins from red onions samples. This highlights how important it is to support any method for the extraction of compounds from natural matrices on an appropriate experimental design. The two methods developed were confirmed to present high repeatability and intermediate precision (RSD < 5%), as well as to require rather short extraction times to achieve good yields. Finally, a multi-response optimization of the two responses, TA and TPC, was carried out, and the resulting UAE method was successfully applied to an assortment of onion varieties. The extraction method was proven to be adequate for the production of extracts from a number of onion varieties with disparate chemical composition. The different extracts were analyzed and high phenolic compounds and anthocyanins contents, as well as good antioxidant activity, were detected.

## Figures and Tables

**Table 2:** Total phenolic compounds and total anthocyanins contents determined by the experiments and predicted values based on the Box–Behnken design.

Run	Factors						Responses			
	*X*_1_	*X*_2_	*X*_3_	*X*_4_	*X*_5_	*X*_6_	*Y_TPC_* (mg g^−1^)		*Y_TA_* (mg g^−1^)	
							Experimental	Predicted	Experimental	Predicted
1	0	0	−1	0	−1	−1	4.1767	3.5242	2.0668	2.0057
2	0	0	1	0	−1	−1	2.9561	3.2168	1.9910	2.0681
3	0	0	−1	0	1	−1	3.2035	3.0330	1.7513	1.5964
4	0	0	1	0	1	−1	2.9146	2.7132	1.3774	1.3945
5	0	0	−1	0	−1	1	2.2442	2.7397	1.8422	1.8561
6	0	0	1	0	−1	1	2.6180	2.4945	1.9246	2.0485
7	0	0	−1	0	1	1	3.0296	3.0630	1.6633	1.6171
8	0	0	1	0	1	1	2.4469	2.8053	1.5150	1.5452
9	0	−1	0	−1	−1	0	2.7012	2.9196	1.9020	2.0085
10	0	1	0	−1	−1	0	2.5148	2.6226	2.1472	1.9832
11	0	−1	0	1	−1	0	2.4628	2.7319	1.7641	1.9643
12	0	1	0	1	−1	0	2.7902	2.3765	2.1085	1.9085
13	0	−1	0	−1	1	0	2.3645	2.5574	1.3898	1.4864
14	0	1	0	−1	1	0	2.5099	2.4617	1.5738	1.4770
15	0	−1	0	1	1	0	3.0411	2.7125	1.4975	1.5580
16	0	1	0	1	1	0	2.5560	2.5584	1.5213	1.5182
17	−1	0	−1	−1	0	0	4.7011	4.7646	1.3071	1.4164
18	1	0	−1	−1	0	0	2.8642	3.0163	0.3299	0.4692
19	−1	0	1	−1	0	0	2.6618	2.4707	1.6671	1.4487
20	1	0	1	−1	0	0	3.3118	3.3064	0.2508	0.4251
21	−1	0	−1	1	0	0	2.7490	3.3144	1.5527	1.3750
22	1	0	−1	1	0	0	3.3056	2.9367	0.2837	0.5053
23	−1	0	1	1	0	0	2.0515	2.4593	1.5521	1.4096
24	1	0	1	1	0	0	5.2890	4.6655	0.5696	0.4635
25	0	−1	−1	0	0	−1	3.6635	3.7722	1.5621	1.5809
26	0	1	−1	0	0	−1	3.9095	4.0100	1.6094	1.6217
27	0	−1	1	0	0	−1	3.2906	3.1606	1.4842	1.5197
28	0	1	1	0	0	−1	4.1254	3.9944	1.5655	1.5434
29	0	−1	−1	0	0	1	4.3195	4.1564	1.5901	1.5813
30	0	1	−1	0	0	1	3.0355	2.8714	1.5588	1.4924
31	0	−1	1	0	0	1	3.4133	3.6069	1.6314	1.6501
32	0	1	1	0	0	1	2.7325	2.9179	1.5319	1.5441
33	−1	−1	0	0	−1	0	2.7751	2.5609	1.9114	1.9364
34	1	−1	0	0	−1	0	3.3414	3.0735	1.4900	1.0493
35	−1	1	0	0	−1	0	2.7997	3.0478	2.1634	2.3055
36	1	1	0	0	−1	0	1.8618	1.9341	0.4220	0.5990
37	−1	−1	0	0	1	0	1.6920	1.8405	1.1957	1.1220
38	1	−1	0	0	1	0	3.4394	3.4122	0.9739	0.9352
39	−1	1	0	0	1	0	2.4817	2.5288	1.1699	1.5071
40	1	1	0	0	1	0	2.4807	2.4741	0.6292	0.5008
41	−1	0	0	−1	0	−1	2.8317	2.8012	1.3718	1.3445
42	1	0	0	−1	0	−1	1.9561	2.1494	0.3941	0.3867
43	−1	0	0	1	0	−1	2.1085	2.0093	1.2244	1.2897
44	1	0	0	1	0	−1	1.9760	2.7280	0.3625	0.4094
45	−1	0	0	−1	0	1	2.3904	2.1984	1.4082	1.3581
46	1	0	0	−1	0	1	2.3983	1.9376	0.4072	0.3451
47	−1	0	0	1	0	1	2.2821	1.5288	1.3218	1.3324
48	1	0	0	1	0	1	2.0481	2.6386	0.3728	0.3969
49	0	0	0	0	0	0	2.3484	2.5702	1.7592	1.7190
50	0	0	0	0	0	0	2.7464	2.5702	1.7587	1.7190
51	0	0	0	0	0	0	2.4622	2.5702	1.7139	1.7190
52	0	0	0	0	0	0	2.6377	2.5702	1.7742	1.7190
53	0	0	0	0	0	0	2.6370	2.5702	1.6840	1.7190
54	0	0	0	0	0	0	2.5892	2.5702	1.6238	1.7190

**Table 3:** ANOVA of the quadratic model adjusted to the extraction of total phenolic compounds from red onion.

Source	Source	Coefficient	Sum of Squares	df	Mean Square	*F*-Value	*p*-Value
Model		2.57	22.39	27	0.83	4.54	0
A—MeOH	*X*_1_	0.11	0.31	1	0.31	1.72	0.2
B—Temperature	*X*_2_	−0.11	0.31	1	0.31	1.67	0.21
C—Amplitude	*X*_3_	−0.14	0.48	1	0.48	2.62	0.12
D—Cycle	*X*_4_	−0.02	0.01	1	0.01	0.07	0.8
E—pH	*X*_5_	−0.05	0.05	1	0.05	0.27	0.61
F—Ratio	*X*_6_	−0.17	0.72	1	0.72	3.94	0.06
AB	*X*_1_ *X*_2_	−0.41	1.32	1	1.32	7.24	0.01
AC	*X*_1_ *X*_3_	0.65	3.34	1	3.34	18.27	0
AD	*X*_1_ *X*_4_	0.34	1.88	1	1.88	10.28	0
AE	*X*_1_ *X*_5_	0.26	0.56	1	0.56	3.07	0.09
AF	*X*_1_ *X*_6_	0.1	0.08	1	0.08	0.42	0.52
BC	*X*_2_ *X*_3_	0.15	0.18	1	0.18	0.97	0.33
BD	*X*_2_ *X*_4_	−0.01	0	1	0	0.01	0.92
BE	*X*_2_ *X*_5_	0.05	0.04	1	0.04	0.22	0.64
BF	*X*_2_ *X*_6_	−0.38	1.16	1	1.16	6.35	0.02
CD	*X*_3_ *X*_4_	0.36	1.04	1	1.04	5.67	0.02
CE	*X*_3_ *X*_5_	−0.00	0	1	0	0	0.98
CF	*X*_3_ *X*_6_	0.02	0	1	0	0.02	0.89
DE	*X*_4_ *X*_5_	0.09	0.06	1	0.06	0.32	0.58
DF	*X*_4_ *X*_6_	0.03	0.01	1	0.01	0.04	0.84
EF	*X*_5_ *X*_6_	0.2	0.33	1	0.33	1.82	0.19
A^2^	*X*_1_^2^	−0.04	0.02	1	0.02	0.12	0.73
B^2^	*X*_2_^2^	0.35	1.25	1	1.25	6.85	0.01
C^2^	*X*_3_^2^	0.88	7.97	1	7.97	43.61	<0.0001
D^2^	*X*_4_^2^	−0.04	0.01	1	0.01	0.08	0.78
E^2^	*X*_5_^2^	−0.26	0.72	1	0.72	3.92	0.06
F^2^	*X*_6_^2^	−0.24	0.58	1	0.58	3.18	0.09
Residual			4.75	26	0.18		
Lack of Fit			4.65	21	0.22	10.93	0.01
Pure Error			0.1	5	0.02		
Cor Total			27.14	53			

**Table 4:** ANOVA of the quadratic model adjusted for the extraction of total anthocyanins from red onion.

Source	Source	Coefficient	Sum of Squares	df	Mean Square	*F*-Value	*p*-Value
Model		1.72	14.37	27	0.53	16.54	<0.0001
A—MeOH	*X*_1_	−0.47	5.38	1	5.38	167.14	<0.0001
B—Temperature	*X*_2_	−0.02	0.01	1	0.01	0.2	0.66
C—Amplitude	*X*_3_	0	0	1	0	0	0.95
D—Cycle	*X*_4_	0	0	1	0	0	0.98
E—pH	*X*_5_	−0.23	1.25	1	1.25	38.83	<0.0001
F—Ratio	*X*_6_	0	0	1	0	0	0.99
AB	*X*_1_ *X*_2_	−0.20	0.34	1	0.34	10.44	0
AC	*X*_1_ *X*_3_	−0.02	0	1	0	0.09	0.77
AD	*X*_1_ *X*_4_	0.02	0.01	1	0.01	0.19	0.67
AE	*X*_1_ *X*_5_	0.18	0.25	1	0.25	7.62	0.01
AF	*X*_1_ *X*_6_	−0.01	0	1	0	0.05	0.83
BC	*X*_2_ *X*_3_	0	0	1	0	0	0.95
BD	*X*_2_ *X*_4_	−0.01	0	1	0	0.01	0.91
BE	*X*_2_ *X*_5_	0	0	1	0	0.01	0.93
BF	*X*_2_ *X*_6_	−0.03	0.01	1	0.01	0.26	0.61
CD	*X*_3_ *X*_4_	0	0	1	0	0	0.99
CE	*X*_3_ *X*_5_	−0.07	0.03	1	0.03	1.09	0.31
CF	*X*_3_ *X*_6_	0.03	0.02	1	0.02	0.53	0.48
DE	*X*_4_ *X*_5_	0.03	0.01	1	0.01	0.21	0.65
DF	*X*_4_ *X*_6_	0.01	0	1	0	0.01	0.91
EF	*X*_5_ *X*_6_	0.04	0.01	1	0.01	0.45	0.51
A^2^	*X*_1_^2^	−0.63	4.02	1	4.02	124.94	<0.0001
B^2^	*X*_2_^2^	−0.02	0.01	1	0.01	0.19	0.66
C^2^	*X*_3_^2^	−0.02	0.01	1	0.01	0.17	0.68
D^2^	*X*_4_^2^	−0.13	0.18	1	0.18	5.53	0.03
E^2^	*X*_5_^2^	0.18	0.32	1	0.32	9.81	0
F^2^	*X*_6_^2^	−0.10	0.11	1	0.11	3.49	0.07
Residual			0.84	26	0.03		
Lack of Fit			0.82	21	0.04	11.79	0.01
Pure Error			0.02	5	0		
Cor Total			15.21	53			

**Table 5:** Separate and simultaneous optimal conditions for the extraction of total phenolic compounds and total anthocyanins.

Factor	Total Phenolic Compounds	Total Anthocyanins	Multi-Response
%MeOH	53	57	50
Temperature (°C)	60	60	53
Amplitude (%)	30.1	89.9	30
Cycle (s)	0.43	0.64	0.4
pH	2.6	2	2
Ratio (g mL^−1^)	0.2:11	0.2:14.9	0.2:14
Result (mg g^−1^) ± SD (*n* = 3)	7.30 ± 0.015	2.49 ± 0.053	7.23 ± 0.034 (TPC); 2.280 ± 0.081 (TA)

**Table 6:** Quantification of total phenolic compounds, total anthocyanins, and antioxidant activity (*n* = 3) extracted by the multi-response developed UAE method to an assortment of onion varieties.

Onion Type	Peak 1 (mg g^−1^)	Peak 2 (mg g^−1^)	Peak 3 (mg g^−1^)	Peak 4 (mg g^−1^)	Peak 5 (mg g^−1^)	Peak 6 (mg g^−1^)	Peak 7 (mg g^−1^)	Peak 8 (mg g^−1^)	Peak 9 (mg g^−1^)	TA (mg g^−1^)	CFT (mg g^−1^)	Antioxidant Activity (mg g^−1^)
Spring white onion I	-	-	-	-	-	-	-	-	-	-	6.01 ± 0.09	4.64 ± 0.13
French white onion	-	-	-	-	-	-	-	-	-	-	6.71 ± 0.18	4.46 ± 0.15
Sweet white onion I	-	-	-	-	-	-	-	-	-	-	2.98 ± 0.04	3.49 ± 0.06
Spring white onion II	-	-	-	-	-	-	-	-	-	-	4.34 ± 0.02	3.26 ± 0.76
Sweet white onion II	-	-	-	-	-	-	-	-	-	-	2.74 ± 0.00	3.72 ± 0.16
CYO white onion	-	-	-	-	-	-	-	-	-	-	4.84 ± 0.04	6.92 ± 0.12
Sweet white onion III	-	-	-	-	-	-	-	-	-	-	5.40 ± 0.12	4.88 ± 0.98
White onion	-	-	-	-	-	-	-	-	-	-	3.31 ± 0.07	5.63 ± 0.31
Babosa white onion	-	-	-	-	-	-	-	-	-	-	5.78 ± 0.17	5.91 ± 0.10
Sweet white onion IV	-	-	-	-	-	-	-	-	-	-	3.30 ± 0.02	2.93 ± 0.03
Fuentes white onion	-	-	-	-	-	-	-	-	-	-	3.64 ± 0.01	4.07 ± 0.32
Yellow onion I	-	-	-	-	-	-	-	-	-	-	5.41 ± 0.17	5.41 ± 0.34
Yellow onion II	-	-	-	-	-	-	-	-	-	-	3.56 ± 0.03	6.14 ± 0.63
Yellow onion III	-	-	-	-	-	-	-	-	-	-	3.53 ± 0.02	6.15 ± 0.07
Yellow onion IV	-	-	-	-	-	-	-	-	-	-	5.00 ± 0.00	3.93 ± 0.10
Yellow onion V	-	-	-	-	-	-	-	-	-	-	5.61 ± 0.11	3.41 ± 0.16
Purple onion	0.43 ± 0.00	0.19 ± 0.00	0.04 ± 0.00	0.02 ± 0.00	0.04 ± 0.00	0.98 ± 0.02	0.27 ± 0.06	0.09 ± 0.00	0.02 ± 0.00	2.28 ± 0.05	7.97 ± 0.03	5.96 ± 0.01
Red onion I	0.20 ± 0.00	0.19 ± 0.00	0.03 ± 0.00	0.02 ± 0.00	0.03 ± 0.00	0.33 ± 0.01	0.23 ± 0.01	0.09 ± 0.00	0.04 ± 0.00	1.36 ± 0.03	6.70 ± 0.08	5.14 ± 0.16
Red label onion	0.24 ± 0.00	0.20 ± 0.01	0.04 ± 0.00	0.02 ± 0.00	0.03 ± 0.01	0.34 ± 0.00	0.24 ± 0.02	0.12 ± 0.01	0.05 ± 0.00	1.46 ± 0.04	8.01 ± 0.12	6.03 ± 0.01
Red onion II	0.53 ± 0.02	0.27 ± 0.01	0.02 ± 0.00	0.05 ± 0.0	0.06 ± 0.01	0.66 ± 0.07	0.34 ± 0.03	0.17 ± 0.01	0.06 ± 0.00	2.39 ± 0.05	7.23 ± 0.08	5.35 ± 0.00
Red onion III	0.31 ± 0.02	0.16 ± 0.01	0.05 ± 0.00	0.03 ± 0.0	0.04 ± 0.00	0.54 ± 0.03	0.23 ± 0.01	0.22 ± 0.00	0.07 ± 0.00	1.64 ± 0.05	6.52 ± 0.08	7.03 ± 0.67
Red onion IV	0.10 ± 0.01	0.09 ± 0.01	0.04 ± 0.00	0.03 ± 0.00	0.23 ± 0.00	0.07 ± 0.00	0.12 ± 0.00	0.03 ± 0.00	0.02 ± 0.00	0.93 ± 0.01	4.54 ± 0.05	7.25 ± 0.12
Purple onion II	0.10 ± 0.00	0.17 ± 0.00	0.04 ± 0.00	0.03 ± 0.00	0.04 ± 0.00	0.46 ± 0.01	0.38 ± 0.00	0.07 ± 0.00	0.03 ± 0.00	1.54 ± 0.02	4.30 ± 0.00	6.43 ± 0.01
Figueres Onion	0.02 ± 00	0.03 ± 0.0	0.02 ± 0.00	0.01 ± 0.0	0.05 ± 0.02	0.02 ± 0.01	0.06 ± 0.02	0.03 ± 0.01	0.02 ± 0.00	0.59 ± 0.00	7.50 ± 0.29	7.10 ± 0.53

## Data Availability

The data presented in this study are contained within the article or Supplementary Material.

## Supplementary Materials

The following are available online at https://www.mdpi.com/article/10.3390/antiox10111755/s1, Figure S1: Information about anthocyanins identified in red onion. Figure S2: MS spectra and structure of the nine anthocyanins identified in onion bulb: (**a**) cyanidin 3-*O*-glucoside; (**b**) cyanidin 3-O-laminaribioside; (**c**) cyanidin 3-*O*-(3″-malonylglucoside); (**d**) peonidin 3-*O*-glucoside; (**e**) delphinidin 3,5-*O*-diglucoside; (**f**) cyanidin 3-*O*-(6″-malonylglucoside); (**g**) cyanidin 3-*O*-(6″-malonyl-laminaribioside); (**h**) peonidin 3-*O*-malonylglucoside; and (**i**) delphinidin 3-*O*-glucoside. The molecular ion is framed in black and the fragments in different colors. Table S1: Mass spectra information of the nine anthocyanins present in onion bulb.
