# Supplementary material for: Development of Optimized Ultrasound-Assisted Extraction Methods for the Recovery of Total Phenolic Compounds and Anthocyanins from Onion Bulbs
Source: Antioxidants (Basel). 2021 Nov 3;10(11):1755. doi: 10.3390/antiox10111755 (PMC8614850; doi:10.3390/antiox10111755)
Supplement: Supplementary file 1 [file antioxidants-10-01755-s001.zip › antioxidants-1432315-supplementary.pdf]

Article

Development of an Optimized Ultrasound-Assisted Extraction Methods for the Recovery of Total Phenolic Compounds and Anthocyanins from Onion Bulbs

Ana V. González-de-Peredo, Mercedes Vázquez-Espinosa, Estrella Espada-Bellido, Marta Ferreiro-González, Ceferino Carrera, Gerardo F. Barbero and Miguel Palma

SUPPLEMENTARY MATERIAL

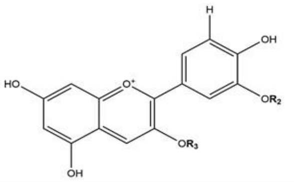

Anthocyanins present in onion	Molecular formula	R_2	R_3
Cyanidin 3-*O*-glucoside	$C_{21}H_{21}O_{11}^{+}$	H	$C_6H_{11}0_5$
Cyanidin 3-*O*-laminaribioside	$C_{27}H_{31}O_{16}^{+}$	H	$C_{12}H_{21}O_{10}$
cyanidin 3-*O*-(3″-malonylglucoside)	$C_{24}H_{23}O_{14}^{+}$	H	$C_9H_{13}O_8$
Peonidin 3-*O*-glucoside	$C_{22}H_{23}O_{11}^{+}$	CH_3	$C_6H_{11}0_5$
Cyanidin 3-*O*-(6″-malonylglucoside)	$C_{24}H_{23}O_{14}^{+}$	H	$C_9H_{13}O_8$
Cyanidin 3-*O*-(6″-malonyl-laminaribioside)	$C_{30}H_{33}O_{19}^{+}$	H	$C_{15}H_{23}O_{13}$
Peonidin 3-*O*-(6"-malonylglucoside)	$C_{25}H_{25}O_{14}^{+}$	CH_3	$C_9H_{13}O_8$

-O-Glucoside	-O-laminaribioside	-O-3′′-malonylglucoside	-O-6′′-malonylglucoside	O-6′′-malonyl-laminaribioside
-O-$C_6H_{11}0_5$	-O-$C_{12}H_{21}O_{10}$	-O-$C_9H_{13}O_8$	-O-$C_9H_{13}O_8$	-O-$C_{15}H_{23}O_{13}$

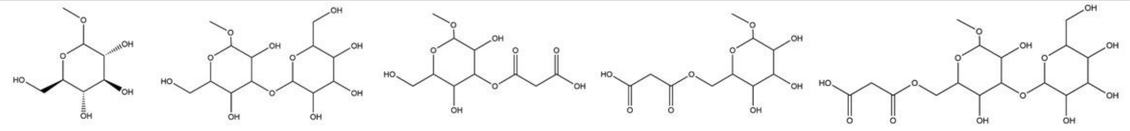

Figure S1. Information about anthocyanins identified in red onion.

Table S1. Mass spectra information of the nine anthocyanins present in onion bulb.

Compounds	Predicted formula	Retention time (min)	*m/z**	Ion Assignment
Cyanidin 3-*O*-glucoside	$C_{21}H_{21}O_{11}^{+}$	3.517	449.1087<br>301.1410<br>287.0557<br>141.9590<br>125.9865	$[M+H]^{+}$<br>$[M+H-C_5H_{10}O_5]^{+}$<br>$[M+H-C_6H_8O_5]^{+}$<br>$[M+H-C_{14}H_{12}O_8]^{+}$<br>$[M+H-C_{15}H_{16}O_8]^{+}$
Cyanidin 3-*O*-laminaribioside	$C_{27}H_{31}O_{16}^{+}$	4.132	611.1641<br>301.1410<br>141.9590<br>125.9865	$[M+H]^{+}$<br>$[M+H-C_{11}H_{18}O_{10}]^{+}$<br>$[M+H-C_{20}H_{22}O_{13}]^{+}$<br>$[M+H-C_{21}H_{26}O_{13}]^{+}$
cyanidin 3-*O*-(3″-malonylglucoside)	$C_{24}H_{23}O_{14}^{+}$	4.875	535.1069<br>301.1410<br>141.9590<br>125.9865	$[M+H]^{+}$<br>$[M+H-C_8H_{10}O_8]^{+}$<br>$[M+H-C_{17}H_{14}O_{11}]^{+}$<br>$[M+H-C_{18}H_{18}O_{11}]^{+}$
Peonidin 3-*O*-glucoside	$C_{22}H_{23}O_{11}^{+}$	5.348	463.1251<br>301.1424<br>141.9590<br>125.9865	$[M+H]^{+}$<br>$[M+H-C_6H_{10}O_5]^{+}$<br>$[M+H-C_{15}H_{14}O_8]^{+}$<br>$[M+H-C_{16}H_{18}O_8]^{+}$
Delphinidin 3,5-*O*-diglucoside	$C_{27}H_{31}O_{17}^{+}$	5.721	649.1392<br>627.1572<br>465.1046<br>303.0511	$[M+H+Na]^{+}$<br>$[M+H]^{+}$<br>$[M+H-C_6H_{10}O_5]^{+}$<br>$[M+H-C_{12}H_{20}O_{10}]^{+}$
Cyanidin 3-*O*-(6″-malonylglucoside)	$C_{24}H_{23}O_{14}^{+}$	5.850	535.1104<br>287.0561<br>141.9590<br>125.9865	$[M+H]^{+}$<br>$[M+H-C_9H_{12}O_8]^{+}$<br>$[M+H-C_{17}H_{14}O_{11}]^{+}$<br>$[M+H-C_{18}H_{18}O_{11}]^{+}$
Cyanidin- 3-*O*-(6″-malonyl-laminaribioside)	$C_{30}H_{33}O_{19}^{+}$	6.052	697.1613<br>287.0561<br>141.9590<br>125.9865	$[M+H]^{+}$<br>$[M+H-C_{15}H_{22}O_{13}]^{+}$<br>$[M+H-C_{23}H_{24}O_{16}]^{+}$<br>$[M+H-C_{24}H_{28}O_{16}]^{+}$
Peonidin 3-*O*-malonylglucoside	$C_{25}H_{25}O_{14}^{+}$	6.323	549.1255<br>301.1418<br>141.9590<br>125.9865	$[M+H]^{+}$<br>$[M+H-C_9H_{12}O_8]^{+}$<br>$[M+H-C_{18}H_{16}O_{11}]^{+}$<br>$[M+H-C_{19}H_{20}O_{11}]^{+}$
Delphinidin 3-*O*-glucoside	$C_{21}H_{21}O_{12}^{+}$	6.536	487.0863<br>465.1055<br>303.0508<br>141.9587<br>125.9865	$[M+H+Na]^{+}$<br>$[M+H]^{+}$<br>$[M+H-C_6H_{10}O_5]^{+}$<br>$[M+H-C_{14}H_{12}O_9]^{+}$<br>$[M+H-C_{15}H_{16}O_9]^{+}$

* The fragments identified have been selected based on their greater abundance or greater selectivity (larger *m/z*).

(a)

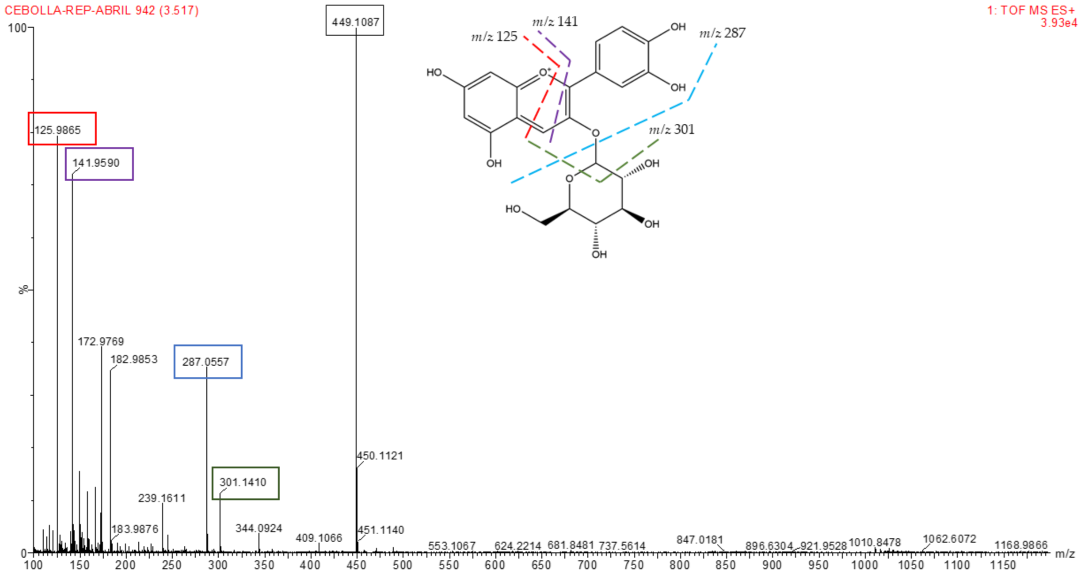
CEBOLLA-REP-ABRIL 942 (3.517)
1: TOF MS ES+
3.93e4
449.1087
125.9865
141.9590
172.9769
182.9853
287.0557
301.1410
450.1121
239.1611
183.9876
344.0924
409.1066
451.1140
553.1067
624.2214
681.8481
737.5614
847.0181
896.6304
921.9528
1010.8478
1062.6072
1168.9866
m/z 125
m/z 141
m/z 287
m/z 301
m/z
%

(b)

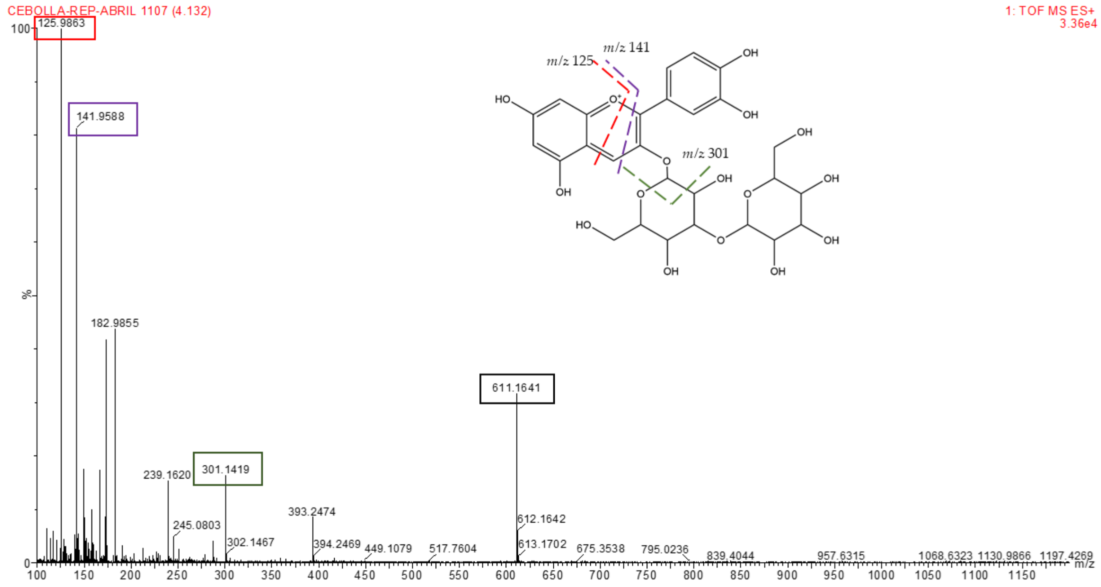
CEBOLLA-REP-ABRIL 1107 (4.132)
1: TOF MS ES+
3.36e4
125.9863
141.9588
182.9855
611.1641
239.1620
301.1419
393.2474
245.0803
302.1467
394.2469
449.1079
517.7604
612.1642
613.1702
675.3538
795.0236
839.4044
957.6315
1068.6323
1130.9866
1197.4269
m/z 125
m/z 141
m/z 301
m/z
%

(c)

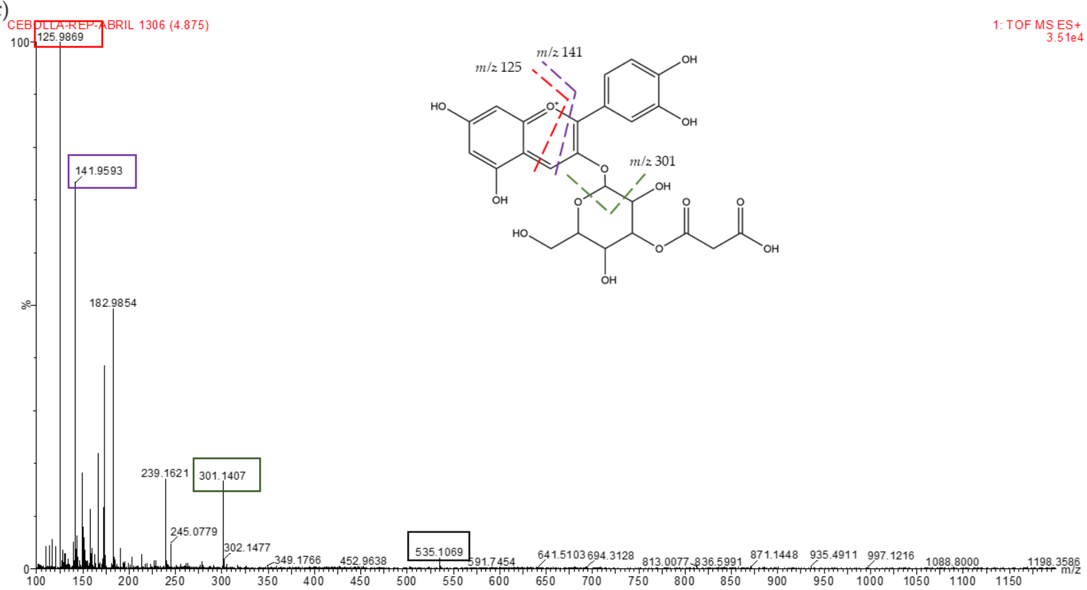
CEBOLLA-REP-ABRIL 1306 (4.875)
1: TOF MS ES+
3.51e4
125.9869
141.9593
182.9854
239.1621
301.1407
245.0779
302.1477
349.1766
452.9638
535.1069
591.7454
641.5103
694.3128
813.0077
836.5991
871.1448
935.4911
997.1216
1088.8000
1198.3586
m/z
%
m/z 125
m/z 141
m/z 301

(d)

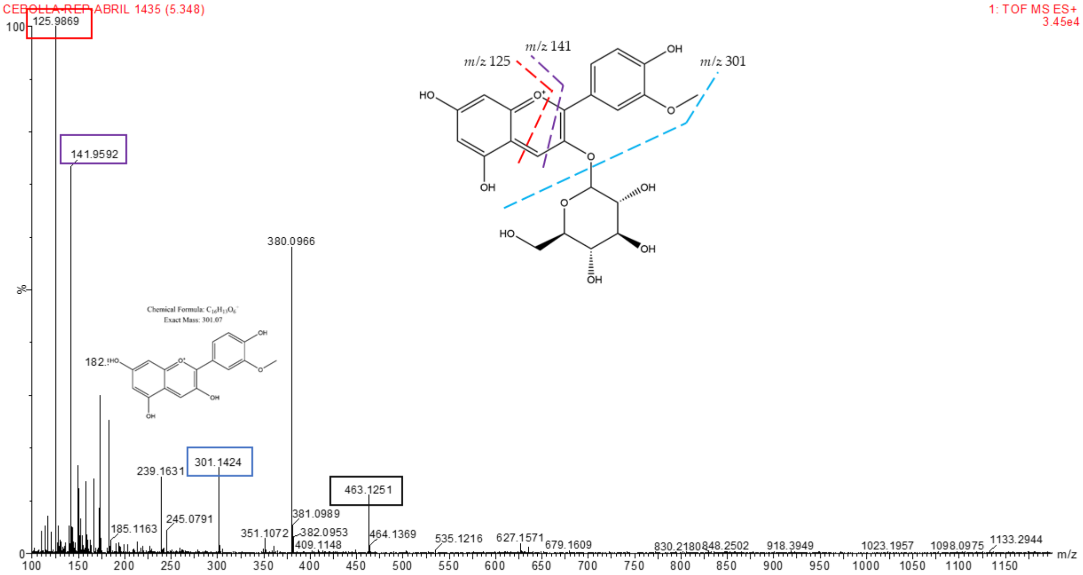
CEBOLLA-REP-ABRIL 1435 (5.348)
1: TOF MS ES+
3.45e4
125.9869
141.9592
380.0966
182.1
239.1631
301.1424
463.1251
381.0989
185.1163
245.0791
351.1072
382.0953
409.1148
464.1369
535.1216
627.1571
679.1609
830.2180
848.2502
918.3949
1023.1957
1098.0975
1133.2944
m/z
%
m/z 125
m/z 141
m/z 301

(e)

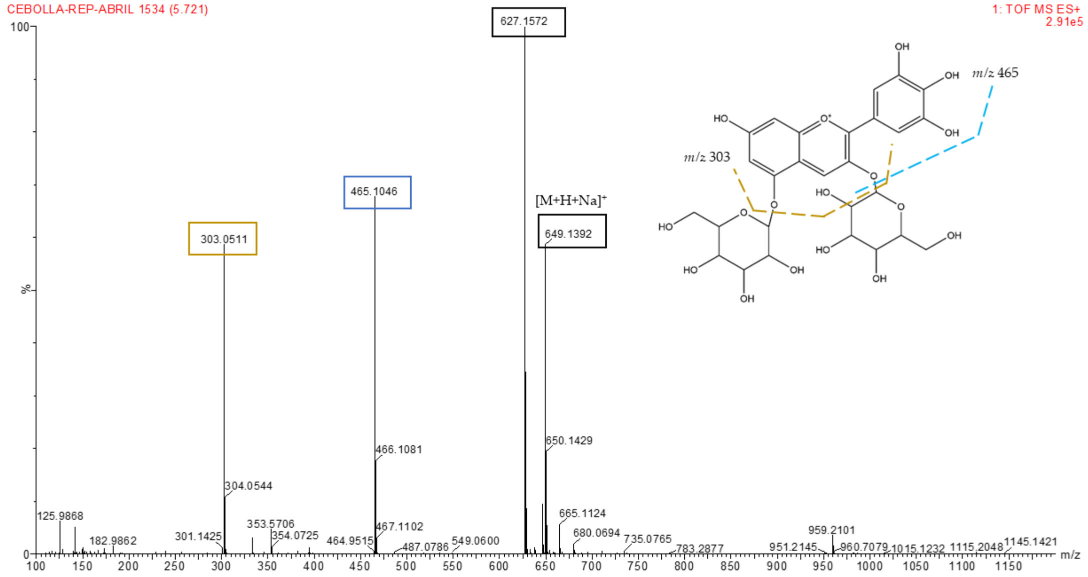
CEBOLLA-REP-ABRIL 1534 (5.721)
1: TOF MS ES+
2.91e5
627.1572
465.1046
303.0511
[M+H+Na]+
649.1392
m/z 465
m/z 303
466.1081
650.1429
304.0544
125.9868
182.9862
301.1425
353.5706
354.0725
464.9515
467.1102
487.0786
549.0600
665.1124
680.0694
735.0765
783.2877
951.2145
959.2101
960.7079
1015.1232
1115.2048
1145.1421
m/z
%

(f)

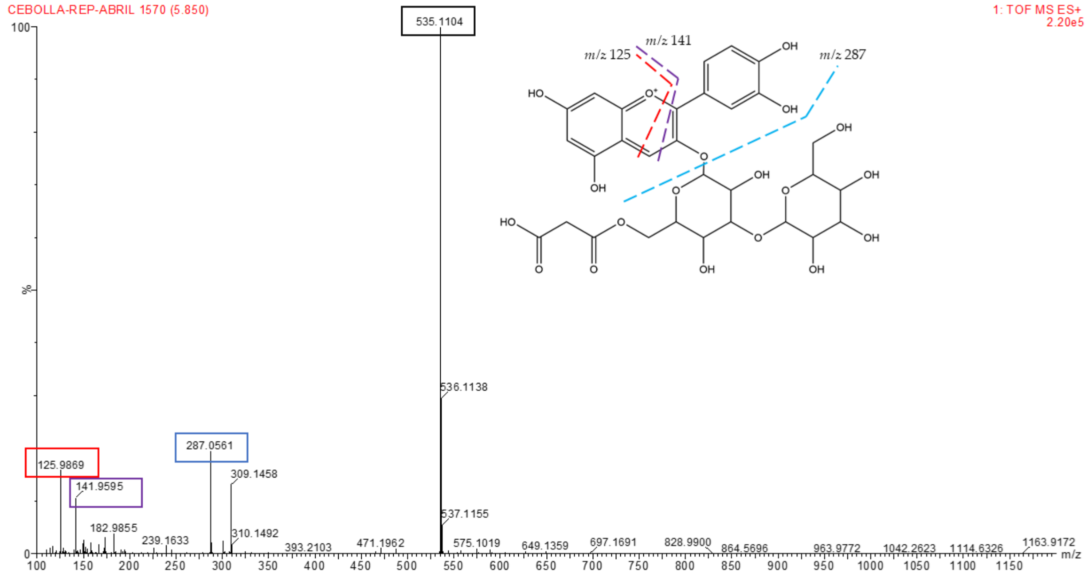
CEBOLLA-REP-ABRIL 1570 (5.850)
1: TOF MS ES+
2.20e5
535.1104
m/z 141
m/z 125
m/z 287
536.1138
125.9869
287.0561
309.1458
141.9595
182.9855
239.1633
310.1492
537.1155
393.2103
471.1962
575.1019
649.1359
697.1691
828.9900
864.5696
963.9772
1042.2623
1114.6326
1163.9172
m/z
%

(g)

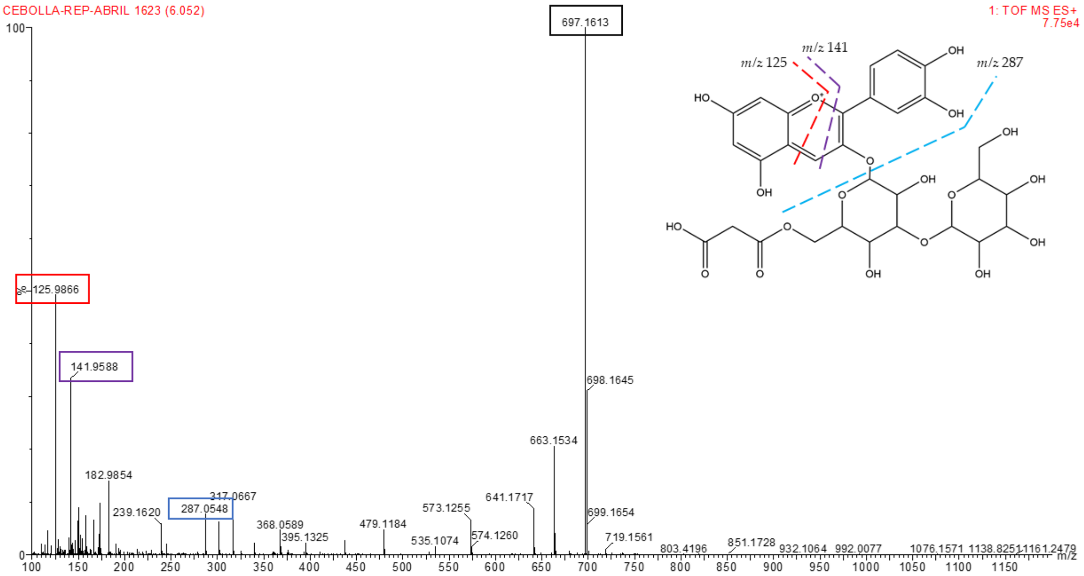

CEBOLLA-REP-ABRIL 1623 (6.052)
1: TOF MS ES+
7.75e4
697.1613
125.9866
141.9588
698.1645
663.1534
182.9854
317.0667
287.0548
239.1620
641.1717
573.1255
699.1654
368.0589
479.1184
395.1325
574.1260
535.1074
719.1561
803.4196
851.1728
932.1064
992.0077
1076.1571
1138.8251
1161.2479
m/z
%
m/z 125
m/z 141
m/z 287

(h)

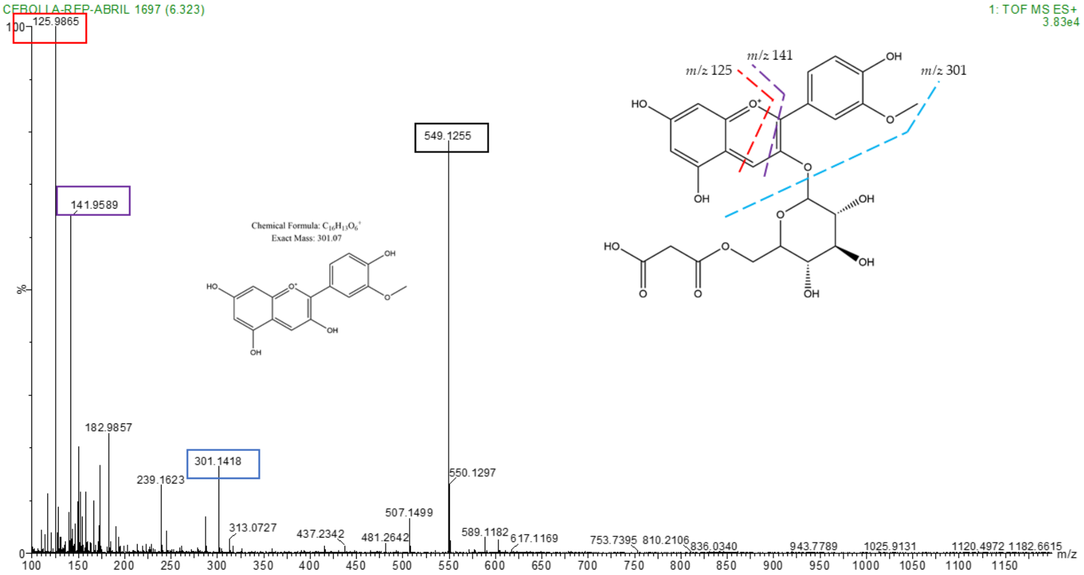

CEBOLLA-REP-ABRIL 1697 (6.323)
1: TOF MS ES+
3.83e4
125.9865
141.9589
549.1255
Chemical Formula: $C_{16}H_{13}O_6^+$
Exact Mass: 301.07
182.9857
301.1418
239.1623
550.1297
507.1499
313.0727
437.2342
481.2642
589.1182
617.1169
753.7395
810.2106
836.0340
943.7789
1025.9131
1120.4972
1182.6615
m/z
%
m/z 125
m/z 141
m/z 301

(i)

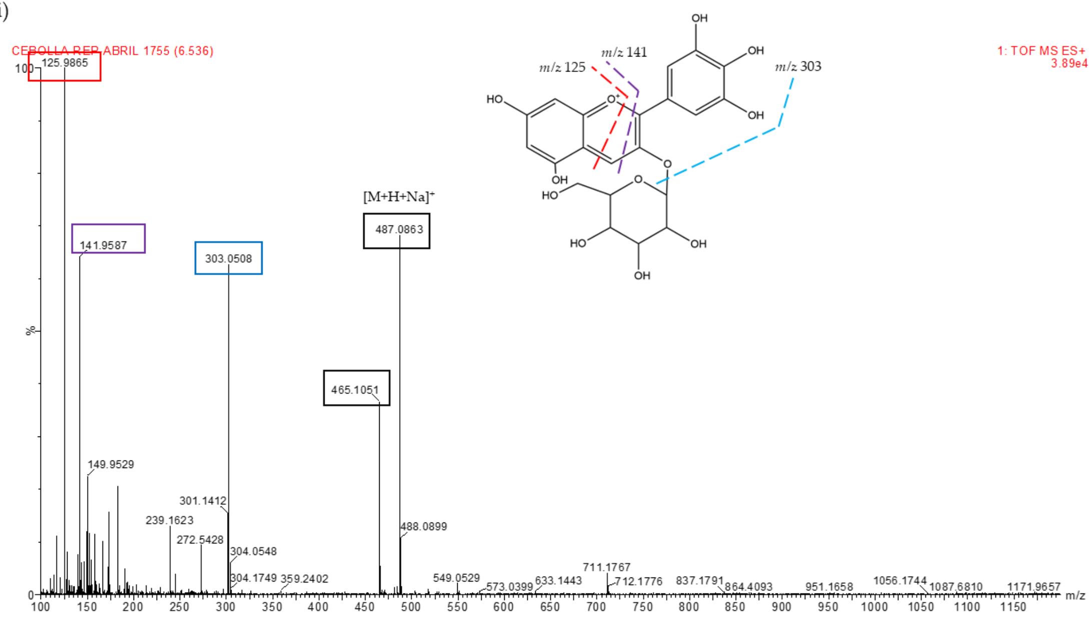

Figure S2. MS spectra and structure of the nine anthocyanins identified in onion bulb: (a) cyanidin 3-*O*-glucoside; (b) cyanidin 3-*O*-laminaribioside; (c) cyanidin 3-*O*-(3″-malonylglucoside); (d) peonidin 3-*O*-glucoside; (e) delphinidin 3,5-*O*-diglucoside; (f) cyanidin 3-*O*-(6″-malonylglucoside); (g) cyanidin 3-*O*-(6″-malonyl-laminaribioside); (h) peonidin 3-*O*-malonylglucoside; (i) delphinidin 3-*O*-glucoside. The molecular ion has been framed in black and the fragments in different colors.
